# Sex-Specific MicroRNAs in Neurovascular Units in Ischemic Stroke

**DOI:** 10.3390/ijms222111888

**Published:** 2021-11-02

**Authors:** Barend W. Florijn, Roel Bijkerk, Nyika D. Kruyt, Anton Jan van Zonneveld, Marieke J. H. Wermer

**Affiliations:** 1 Department of Neurology, Leiden University Medical Center, 2333 ZR Leiden, The Netherlands; n.d.kruyt@lumc.nl (N.D.K.); m.j.h.wermer@lumc.nl (M.J.H.W.); 2 Einthoven Laboratory for Vascular and Regenerative Medicine, Leiden University Medical Center, 2333 ZA Leiden, The Netherlands; r.bijkerk@lumc.nl (R.B.); ajvanzonneveld@lumc.nl (A.J.v.Z.); 3 Department of Internal Medicine (Nephrology), Leiden University Medical Center, 2333 ZA Leiden, The Netherlands

**Keywords:** microRNA, stroke, women, neurovascular unit

## Abstract

Accumulating evidence pinpoints sex differences in stroke incidence, etiology and outcome. Therefore, more understanding of the sex-specific mechanisms that lead to ischemic stroke and aggravation of secondary damage after stroke is needed. Our current mechanistic understanding of cerebral ischemia states that endothelial quiescence in neurovascular units (NVUs) is a major physiological parameter affecting the cellular response to neuron, astrocyte and vascular smooth muscle cell (VSMC) injury. Although a hallmark of the response to injury in these cells is transcriptional activation, noncoding RNAs such as microRNAs exhibit cell-type and context dependent regulation of gene expression at the post-transcriptional level. This review assesses whether sex-specific microRNA expression (either derived from X-chromosome loci following incomplete X-chromosome inactivation or regulated by estrogen in their biogenesis) in these cells controls NVU quiescence, and as such, could differentiate stroke pathophysiology in women compared to men. Their adverse expression was found to decrease tight junction affinity in endothelial cells and activate VSMC proliferation, while their regulation of paracrine astrocyte signaling was shown to neutralize sex-specific apoptotic pathways in neurons. As such, these microRNAs have cell type-specific functions in astrocytes and vascular cells which act on one another, thereby affecting the cell viability of neurons. Furthermore, these microRNAs display actual and potential clinical implications as diagnostic and prognostic biomarkers in ischemic stroke and in predicting therapeutic response to antiplatelet therapy. In conclusion, this review improves the current mechanistic understanding of the molecular mechanisms leading to ischemic stroke in women and highlights the clinical promise of sex-specific microRNAs as novel diagnostic biomarkers for (silent) ischemic stroke.

## 1. Introduction

### 1.1. Sex Differences in Stroke

Accumulating evidence suggests that there are sex differences in stroke, in terms of incidence and clinical presentation, etiology, and outcome. Although young women (25–44 year age groups) have a higher stroke incidence (incidence rate ratio 0.87 [95% CI, 0.78–0.98]) than young men (0.70 [95% CI, 0.57–0.86]), this trend is reversed in older age groups, with a higher acute stroke incidence ratio in men [1]. Mechanistic studies into more female-specific trends in acute stroke have demonstrated that compared to men, women also have a different profile of vascular risk factors (more hypertension, atrial fibrillation, heart failure, valvular heart disease and obesity), a different distribution of stroke subtypes (more cardio-embolic stroke) and a different outcome (worse early outcome, more disability, longer hospital stay and higher in-hospital mortality) [2]. Furthermore, acute stroke in women seldom presents typical stroke symptoms like language disturbance and weakness (13.7% versus 9.5%) [3], thereby making women more likely to receive a stroke mimic diagnosis [4,5]. Stroke in women however, is driven by divergent risk factors (atrial fibrillation and cardio-embolism) compared to stroke in men (atherosclerosis and internal carotid artery disease) [6], and several sex-specific risk factors increase this female stroke incidence [7] such as hypertension [8], diabetes mellitus [9,10], preeclampsia [11] and migraine [12]. Given the higher likelihood of disability and worse quality of life following a stroke diagnosis in women, early recognition [5] and better understanding of the sex-specific mechanisms leading to ischemic stroke in women could augment its early detection, which could improve clinical care and treatment standards.

### 1.2. Neurovascular Units and Ischemic Brain Injury

**Figure 1:**
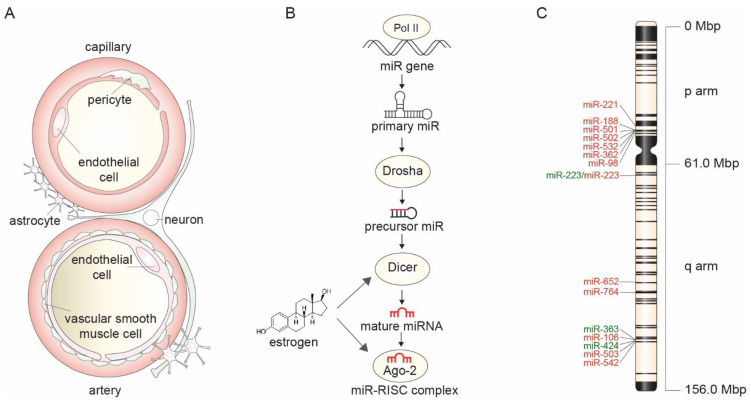
(**A**) The neurovascular unit is a functional unit of neurons, astrocytes, endothelial cells (ECs) and vascular smooth muscle cells (VSMCs) that controls neurovascular coupling, and thereby cerebral blood flow (CBF). (**B**) Estrogen can affect miR transcription and action by controlling the expression of the pre-miR processing protein Dicer, and by increasing Argonaute-2 protein (crucial for miR target recognition and thereby its function) respectively. (**C**) X-chromosome and X-linked miRs involved in the cellular response to injury of neurons, astrocytes, ECs and VSMCs. Red font depicts stroke promoting miRs while green font indicates stroke preventing miRs.

A deeper mechanistic understanding of ischemic brain injury is becoming available with the concept of dysfunctional cell communication in neurovascular units (NVUs). This functional unit consists of neurons, astrocytes, endothelial cells (ECs), pericytes and vascular smooth muscle cells (VSMCs), and controls, among other processes, neurovascular coupling, thereby regulating cerebral blood flow (CBF) (Figure 1A) [13]. Neurovascular coupling is vasomotion reactive to local neural activity, and is regulated following EC signaling to arteriolar VSMCs and capillary pericytes [14]. ECs also secrete trophic factors that protect neurons against oxygen-glucose deprivation (OGD) [15], while astrocytes produce extra cellular matrix (ECM) factors that promote EC quiescence [16] and blood-brain barrier (BBB) integrity [17]. Interestingly, experimental studies into the sex-specific behavior of NVUs after stroke have demonstrated a sex-specific cellular response to injury to ECs [18], astrocytes [19,20] and neurons [21]. This illustrates the potential for sex-specific dysfunctional NVU cell–cell communication [22] to drive ischemic injury differently in women and men [23].

### 1.3. Sex-Specific MicroRNA Expression in Neurovascular Units

Increasing evidence suggests that the cellular response to ischemic injury is largely regulated at the post-transcriptional level, involving microRNAs [24] which regulate target messenger RNA (mRNA) expression based on complementary base pairing at the mRNA 3′-untranslated region [25]. Two main driving factors can influence sex-specific microRNA expression. First, the X-chromosome encodes 118 microRNAs [26], and several X-chromosome located (X-linked) microRNAs can escape X-chromosome inactivation [27], resulting in their higher expression in women [28]. Second, numerous gene promoters include estrogen-responsive elements (EREs) [29], whereby estrogen binding or release steers miR expression [30]. Previously, we reviewed the question of whether these mechanisms result in sex-specific microRNA expression within female microvascular cells [31]. We subsequently demonstrated that their sex-specific expression instigates a different clinical outcome in women compared to men, such as a higher prevalence of microvascular injury (defined by elevated angiopoietin-2 levels) following diabetes mellitus [32]. Lastly, we described the regulatory role of angiogenic microRNAs in cerebral small vessel disease [33]. Consistent with these results, we hypothesized that sex-specific microRNAs could improve our understanding of the molecular mechanisms leading to ischemic stroke in women, and may be of actual and potential clinical relevance as diagnostic and prognostic biomarkers, as well as for predicting therapeutic response to antiplatelet therapy.

### 1.4. Purpose of the Study

This narrative review describes the sex-specific expression of microRNAs in NVUs and their actual and potential clinical application. We first provide an overview of the mechanisms of sex-specific microRNA expression. Second, we demonstrate that these microRNAs function as a post-transcriptional regulatory network of a sex-specific cellular response to injury in cerebral ECs, VSMCs and neurons. This either promotes or prevents dysfunctional cell–cell communication in NVUs leading to ischemic stroke. Lastly, we describe how these microRNAs could function as a diagnostic and prognostic biomarker in stroke, and aid in predicting therapeutic response to antiplatelet therapy.

## 2. Mechanisms of Sex-Specific MicroRNA Expression

### 2.1. X-Chromosome Mosaicism and Its Effect on MicroRNA Expression

X-chromosome gene dosage disequilibrium (resulting from two X-chromosome copies in female somatic cells) is prevented by the neutralization of one X-chromosome by random X-chromosome inactivation (XCI) during embryonic development [34]. XCI is coordinated by the long noncoding RNA (lncRNA) ‘X-inactive specific transcript’ (XIST), and starts with X chromosome counting, random X chromosome choice [35] and subsequent binding of polycomb repressive complexes that induce methylation and silencing of the genes located on the X chromosome [36]. These steps are carried out in the peri-implantation embryo of the 10–20 cell epiblast lineage and should be maintained in all NVU somatic cells. Nonetheless, female somatic cells are mosaic cells because of heterogenous X-linked gene expression, since 15% of X-linked genes permanently escape XCI, while 10% display variable expression patterns as a result of incomplete XCI [28]. This could contribute to the molecular mechanisms that underlie the female-specific cardiovascular etiology as observed in stroke, because stroke risk factors like obesity [37] and hypertension [38] are associated with a higher X-chromosome gene dosage and a more pronounced cellular response to EC and VSMC injury within the microvasculature in women [31].

Collectively, these studies indicate that the incomplete silencing of X-chromosomes in NVU cells can promote elevated X-linked microRNA expression in NVUs (Figure 1C), thereby altering their cellular response to injury. Whether this process initiates EC activation and NVU dysfunction, which may eventually lead to cerebral small vessel disease (SVD), as recently observed in a rat SVD model [39], should be further investigated. These studies should employ unbiased single-cell RNA sequencing methods followed by single cell ablation experiments to decipher the subsequent cellular response after incomplete inactivation of X-chromosome genes in NVUs.

### 2.2. Estrogen Control of MicroRNA Biogenesis

Experimental studies in noncerebral cells (MCF-7 breast cancer cells, which contain abnormal gene expression profiles and exhibit a deregulated cell cycle control) have demonstrated that estrogen receptor (ER) binding to estrogen response elements (EREs) in gene promotors, drives microRNA expression thereby regulating multiple downstream target genes [40]. Furthermore, several components of the microRNA transcription and processing machinery itself are regulated by estrogen because estrogen enhances levels of the Dicer protein [41] and the RISC complex catalytic subunit, Argonaute-2 (Ago-2) [42] (Figure 1). Interestingly, when neurons are exposed to estrogen in vitro and in vivo, this alters their microRNA expression levels. This was demonstrated in a study that assessed estrogen-regulated microRNA-(miR)-30b levels in cultured glioblastoma cells and neurons derived from mouse frontal cortex and hippocampal tissue as well as post-mortem prefrontal cortex tissue from schizophrenia patients (*n* = 30). These miR-30b levels increased 2-fold in cultured glioblastoma cells exposed to estrogen and were present in higher levels in female mouse brain compared to male mouse brain [43]. However, precursor forms of miR-30b were not significantly different in the human subjects (suggesting no estrogen effect on miR biogenesis) while altered Dicer or Argonaute protein levels in response to estrogen were not assessed in vitro or in mice [43]. In humans, women with schizophrenia displayed a significant reduction in miR-30b levels compared to healthy control women, while miR-30b levels between women and men were not significantly different [43]. This could suggest that miR expression develops differently between men and women following neuronal estrogen exposure. Interestingly, in human ECs, estrogen exposure was found to increase both pri-miR-126 and mature miR-126-3p expression in an ERα-dependent manner, suggesting that estrogen controls microRNA biogenesis in vascular cells [43]. Whether these results indicate that estrogen regulates microRNA control sex-specific neurovascular coupling in women and men should be assessed in future studies [44].

Taken together, these studies on estrogen-regulated miR biogenesis were predominantly carried out in MC7- and glioblastoma cell cultures, and thus, provide no definite answers to the question of whether estrogen regulates each step in microRNA biogenesis (Figure 1B). However, estrogen regulates microRNA expression in NVUs as well, as was demonstrated in cultured neurons [43] and in vivo in astrocytes from mice [45]. This could contribute to the molecular mechanisms of the NVU cellular response to a female-specific cardiovascular etiology in stroke.

## 3. Stroke Promoting Mechanisms

### 3.1. Sex-Specific MicroRNAs and Blood Brain Barrier Integrity

A striking pathophysiological feature of ischemic stroke is BBB disruption [46]. The highly polarized ECs of cerebral blood vessels regulate BBB properties, such as its paracellular barrier function and tight junctions. Tight junctions contain transmembrane proteins (claudin-, occludin- and junctional adhesion proteins), scaffolded by other proteins like the zona occludens proteins that form intercellular connections [14]. This promotes a tight barrier whose maintenance is regulated by other NVU cells like neurons that secrete Wnt ligands [47], astrocytes which produce extracellular matrix (ECM) factors [48] and pericytes which promote endothelial quiescence [49].

Following local ischemia, altered cell-specific metabolomic adaptations perturb BBB function and integrity [50] and immune cells promote neuroinflammation and apoptosis of neurons [51]. This could alter NVU function differently in women and men given that women display a lower metabolic brain age (relative to their chronological age) compared to men (i.e., less loss of cerebral blood flow and more brain glycolysis at a younger age) [52]. Experimental studies into a different signaling of NVUs in women and men also pinpoint such sex-specific outcomes regarding BBB integrity. For instance, brain microvascular ECs (BMECs), derived from differentiated induced pluripotent stem cell (iPSC) lines from premenopausal women, display an increased barrier strength due to a decreased BBB permeability compared to iPSC-derived BMECs from men [53]. Furthermore, it has been demonstrated that when cerebral ECs are cultured in stroke-like OGD culture models, female ECs display less sensitivity to OGD [54] and endothelin-1 mediated vasoconstriction than male ECs [55]. However, these neuroprotective, sex-specific functions of NVUs in vasomotion are lost following menopause [56]. Therefore, these observations make way for the hypothesis that estrogen-regulated microRNAs may affect cell signaling, and thus NVU function differently in women and men.

Various in vitro cell models have addressed the role of microRNA expression in BBB integrity and function, for instance by investigating TNF-α stimulation of cultured murine brain vascular endothelial (bEnd3) cells. In these cells, this experimental setting significantly increased the expression of the X-linked miR-501-3p [57]. This miR-501 increase was similarly observed in brain white matter lesions from mice, following bilateral common carotid artery stenosis (BCAS) surgery, a model of white matter lesions and BBB breakdown [57]. With higher expression of this microRNA in inflammation and BCAS, a decrease in the expression of tight-junction protein 1 (ZO-1) was seen which led to a decrease in tight junction affinity [57]. Another microRNA derived from the X-chromosome and involved in tight junction affinity following stroke was identified in plasma from acute ischemic stroke patients, which contains high levels of the X-linked miR-503, although no sex differences in its circulating levels were assessed [57]. Also, when mice following middle cerebral artery occlusion (MCAO) were treated with plasma from these acute stroke patients, it promoted EC injury, BBB permeability and increased infarct volume, which were all ameliorated by miR-503 inhibition [58]. Interestingly, in vitro, miR-503 knockdown in human brain microvascular ECs (HBMECs) downregulated about 50% of reactive oxygen species (ROS) production and upregulated about 30% of nitric oxide (NO) generation leading to a higher expression of the pro-survival p-PI3K/Akt/eNOS signaling pathways. Another example of potential deleterious effects on BBB integrity by increased expression of X-linked microRNAs comes from the fact that overexpression of the X-linked miR-424–5p decreased the expression of tight junction proteins ZO-1 and occludin, via increased expression of endophilin-1, a protein that affects BBB permeability by regulating tight junction-related proteins through a process involving the EGFR-ERK and EGFR-JNK signaling pathways [59]. Interestingly, amongst all microRNAs, both miR-424 and miR-503 are predominantly regulated by estrogen [60,61] while miR-503 is known to control female CD4^+^ T cell activation and as such the acute inflammatory response in women [62]. More support for elevated X-linked miR expression and potential sex-specific downstream effects in brain ECs was demonstrated in OGD cultured cerebral ECs which increased miR-106a expression, thereby decreasing cell viability, leading to cell death due to an increased and pro-inflammatory caspase-3 expression [63]. Also, murine cerebrovascular endothelial cells displayed less cell viability upon transient MCA occlusion in C57BL/6 J male mice (*n*  =  24, 8–10 weeks old), a phenotype induced by elevated miR-106a expression [63].

In contrast to these detrimental effects on BBB integrity via increased expression of X-chromosome located microRNAs, their increase may also exhibit NVU protective effects, as was observed for the X-linked miR-98. The expression of this miR is reduced in OGD cultured primary brain microvascular endothelial cells (BMVECs) and in mice following transient MCAO which was found to disrupt BBB integrity. By restoring its expression in experimental studies, EC barrier function improved, BBB breakdown was rescued and diminished infiltration of brain leukocyte influx and neuroinflammation in mice was seen [64]. Interestingly, miR-98 is an estrogen-responsive miR and under estrogen receptor (ER)α-control in breast cancer cells. This estrogen control of miR-98 function is known to regulate local inflammation by decreasing cellular IL-6 gene expression, thereby attenuating the release of proinflammatory cytokines such as TNF-α and IL-1β [41]. Furthermore, the expression of miR-98 is increased in female CD4^+^ T cells which partly controls the immune response at the post-transcriptional level.

Collectively, these studies suggest that differential expression of X-linked microRNAs alters endothelial tight junction expression and thus BBB integrity in response to ischemic injury in stroke-like conditions. The fact that both the X-linked miR-424 and miR-501 regulate the expression of ZO-1 protein, and thereby, EC tight junction function, pinpoints the synergistic function of microRNAs (regulating the same gene) in BBB integrity. As such, this simultaneous repression of ZO-1 protein directly influences the output of functionally related biological pathways (BBB integrity) [65] and thus could provide additional mechanistic insight into sex-specific stroke pathophysiology. Nonetheless, it should be mentioned that these studies were predominantly carried out in murine models of stroke, such as (transient) MCAO or BCAS surgery models. Therefore, additional (human) studies into microRNA expression in stroke in which expression results are stratified for women and men are needed to determine whether the relative overexpression of these X-linked microRNAs is conserved in humans and contributes to female stroke pathophysiology.

### 3.2. Sex-Specific MicroRNAs and Vascular Smooth Muscle Cell Proliferation

VSMCs play a key role in vessel wall remodeling in response to cerebral ischemic injury and neurodegenerative disease. Following ischemia and EC activation, VSMCs switch from a contractile phenotype, that normally coordinates vascular vasodilation and constriction, to a synthetic phenotype that proliferates and migrates [66], and instigates arterial stiffening, atherosclerosis and calcification [67]. Interestingly, several X-linked microRNAs are associated with these steps as upstream regulators of gene expression.

A striking example of X-linked microRNA expression in VSMC activation is miR-362-3p of which plasma levels were significantly decreased in atherosclerotic patients (*n* = 110) compared to control patients (*n* = 84) (although clinical factors in this study, such as sex, were not significantly different in patients versus controls) [68]. Subsequent in vitro studies with this microRNA demonstrated that miR-362-3p mediated overexpression in cultured VSMCs reduced cellular proliferation and migration and induced G1 cell cycle arrest. These effects were mediated via AdamTs1 (a metalloproteinase involved in the development of atherosclerosis) which plasma levels were increased in atherosclerotic patients compared to controls (both mRNA and protein levels) [68]. This suggests that the loss of miR-362-3p promotes proliferations of VSMCs. More X-linked microRNA mediated effects on VSMC proliferation were seen in a study into the effects of miR-532-5p. Levels of this particular miR were decreased in plasma from atherosclerotic patients (*n* = 103) compared to healthy controls (*n* = 77), inversely related to carotid intima-media thickness (CIMT) and displayed a high sensitivity and specificity for atherosclerosis (AUC 0.897) [69]. However, no experimental studies into target gene expression differences were carried out in this study.

In cerebral VSMC calcification, another pathological event in atherosclerosis development, several X-linked microRNAs are involved, while the subsequent differentiation of these cells into osteoblast- and chondrocyte-like cells following microRNA expression changes can occur as well. Studies in VSMCs from aged rats have demonstrated that aged VSMCs have decreased miR-542-3p expression compared to VSMCs from younger rats [70]. However, the RNA used for the experiments in this study was extracted after passage of cells in culture, which is known to affect cellular gene expression. Still, this study identified that overexpressing miR-542-3p in young VSMCs suppressed their osteogenic differentiation induced by β-glycerophosphate (β-GP, an in vitro model of VSMC calcification) via a reduction of bone morphogenetic protein 7 (BMP7, a protein known for inducing osteogenesis and chondrogenesis) expression. More beneficial effects for X-linked microRNA expression in VSMCs were found in human VSMCs treated with oxidized low-density lipoprotein (ox-LDL) and in carotid VSMCs from ApoE^−/−^ C56BL/6J mice (*n* = 5) fed a high-fat diet. In these experimental settings, the X-linked miR-188 decreased in VSMCs from mice fed a high-fat diet compared to controls. As a consequence, the expression of its gene target, fibroblast growth factor 1 (Fgf1) was elevated [71]. Functionally, this microRNA represses the proliferation and migration of VSMCs and promotes their apoptosis by specifically downregulating Fgf1, suggesting that its role is to counteract atherosclerotic pathways [71]. Furthermore, X-linked miR-503-5p also represses VSMC proliferation and migration, and its levels have been found to be decreased in patients with carotid artery stenosis (*n* = 62) compared to healthy controls (*n* = 60) [59]. This particular miR displayed strong diagnostic potential for carotid artery stenosis, with an ROC value of 0.817, a specificity of 79.0% and a sensitivity of 83.3% [72]. Moreover, the experimentally decreased expression in vitro of this microRNA in VSMCs promoted cellular proliferation, suggesting that it indirectly instigates vascular hyperactivity in the setting of carotid artery stenosis. Finally, cell-cell communication from macrophages to VSMCs in atherosclerotic plaques can also proceed following altered X-linked miR expression. This was demonstrated in a study with ox-LDL treated macrophages (THP1-cells) which transfer functional microRNAs via exosomes to VSMCs to promote cell proliferation and repress cell apoptosis. These exosomes from THP-1 cells contained the X-linked miR-106a-3p of their uptake in VSMCs increased the expression of this microRNA in VSMCs [73]. The resultant overexpression of miR-106 promoted cell viability and decreased cellular apoptosis because caspase signaling was decreased in VSMCs [73].

In summary, these studies demonstrate that following carotid artery atherosclerosis, the expression of particular X-linked microRNAs (i.e., miR-188, miR-362, miR-503, miR-532, miR-542) is decreased and promotes VSMC proliferation. Although gene expression results in these studies were not stratified for women and men, these findings could provide a novel molecular mechanism for sex-specific atherosclerotic phenotypes given that extracranial atherosclerosis is less often present in women compared to men [74].

### 3.3. Sex-Specific MicroRNAs and Apoptosis of Neurons

Cellular hypoxia and the loss of glucose and ATP supply following stroke, promote Na+/K+ pump failure, depolarization of neurons and glutamate release. Following an ensuing activation of glutamate receptors like the N-methyl-D-aspartate (NMDA) receptors, cellular excitation due to increased Ca^2+^ influx promotes nitric oxide synthase (nNOS) and calpain I release, thereby increasing cellular reactive oxygen species (ROS) [75]. This increases the permeability of mitochondria and eventually results in cell death by necrosis.

Interestingly microRNAs derived from the X-chromosome were found to play a significant role in regulating multiple sets of functionally related genes that modulate the neuronal effects induced by cellular hypoxia and ischemia (i.e., the regulation of Ca^2+^ influx, cellular oxidative stress and apoptosis). Particular the X-linked miR-223 was found to be elevated in neurons from rats at different time intervals after ischemia (10-fold in cortex neurons after 48 h and 20–30 fold in the striatum 24 h) while its target gene Nckx2, a member 2 of the K^+^-dependent Na^+^/Ca^2+^ exchanger family, was decreased [76]. This protein regulates sodium and calcium homeostasis in ischemic neurons and therefore compensates the altered ionic homeostasis after stroke. Its decrease upon hypoxia suggests that elevated miR-223 affects neuronal outcome negatively, as underscored by the decrease of infarct volume after antimiR-223 treatment which lowered its functional levels. Following the subsequent entrance of Ca^2+^ ions in neurons, X-linked microRNAs also control neuronal apoptosis by regulating cellular oxidative stress and mitochondrial respiration. One study into neuronal mitochondrial hemodynamics after stroke demonstrated that neurons isolated from a rat cerebral ischemia/reperfusion (CI/R) injury model display increased expression of the cerebral NADPH oxidase 2 (Nox2), which promotes ROS production in neurons. This phenomenon coincided with less expression of miR-652, suggesting that this microRNA protects against ischemic events, a conclusion emphasized by systemic injection of miR-652 agomirs, which improved cellular apoptosis and infarction in rats [77]. More evidence for miR-652 regulation of mitochondrial hemodynamics following ischemia was demonstrated in a study in which elevated NR4A2 expression (a transcription factor that regulates mitochondrial homeostasis and neuronal survival) after transient global cerebral ischemia decreased miR-652 expression but increased the mitochondrial E3 ubiquitin ligase 1 (Mul1) protein (known for regulating mitochondrial dynamics following stroke), thereby repressing cellular viability [78].

In vitro stroke models of the aforementioned mechanisms of neuronal cellular stress (like cell culture of neurons in OGD conditions) have demonstrated marked upregulation of the X-linked miRs involved in ischemic cell death (caspase) pathways. A clear example is the elevated expression of the X-linked miR-188-5p in OGD-cultured neurons, the expression of which coincided with lower cell proliferation over time and higher expression of apoptosis markers caspase-3 and caspase-8 [79]. OGD simultaneously increased phosphatase and tensin homolog (Pten), a putative miR-188 target which negatively regulates phosphatidylinositol-3, 4, 5- trisphosphate (Pi3k)/Akt signaling in neurons [80]. Although this study did not describe the specific downstream effectors which regulate these processes, it suggests that the elevated Pten expression makes neurons more sensitive to ischemia-reperfusion injury, as observed by increased apoptosis markers caspase-3 and -8, which suppressed cell viability over time. More evidence for X-linked miR regulation of neuronal apoptosis comes from the fact that miR-502 expression was increased in cultured neurons (a murine HT22 nerve cell line) under inflammatory conditions which led to a decrease in Cdkn1b expression, also known as p27KIP1, which negatively regulates cellular proliferation via cyclin dependent kinases [81]. As a result, more apoptosis of this cell line was observed. Similarly, in human primary neurons, lentivirus-mediated overexpression of the X-linked miR-764 led to a significant repression of Ninj2 (an adhesion molecule with higher expression in neurons and elevated after nerve injury) mRNA and protein expression [82]. The decrease of this particular protein by miR-764 significantly (*p* < 0.05) reduced cell viability, leading to apoptosis of neurons (as determined by MTT assays and TUNEL staining of cells) [83]. More negative effects on the proliferation of neurons and its cellular viability were seen in OGD cultured cortical neurons derived from rats which displayed increased levels of the X-linked miR-223, which were found to suppress cortical neuron proliferation by decreasing type 1 insulin-like growth factor receptor (Igfr1) mRNA and protein expression levels [84]. This protein is a transmembrane receptor tyrosine kinase which is known for regulating the biological activity of insulin growth factor that inhibits cell apoptosis induced by hypoxia. As such, in this study, miR-223 inhibited the proliferation of neurons and their cellular viability after OGD, eventually leading to ischemic brain damage [84].

In conclusion, these studies indicate that the neuronal response to OGD regulates cellular ion balance and proliferation (miR-223), and activates caspase-mediated cell-death pathways (regulated by miR-188-5p) and apoptosis of neurons (miR-502 and miR-764). Because the majority of these studies were performed in mice and rats, additional studies are needed to investigate whether these miRs have a conserved role in NVU homeostasis in humans as well, as was demonstrated for miR-188-5p, of which the altered expression in humans contributes to Alzheimer disease (AD) pathogenesis [85]. Cellular overexpression of these microRNAs in human NVUs, as a result of their double dosage due to incomplete X-chromosome inactivation, could render neurons less viable after ischemia. This could provide novel mechanistic insight into currently known ischemic cell death pathways in neurons which were found to display striking sex-differences. While male neurons exhibit caspase-independent cell death pathways like apoptosis-inducing factor (AIF) and Poly(ADP-ribose) polymerase (PARP) activation (thereby invoking mitochondrial depolarization and cell death) [86], female neurons rely on caspase-mediated cell death pathways [87]. Following this notion, many experimental studies have been conducted aiming at deepening our mechanistic understanding of sex-specific cell death pathways in neurons. Particularly in seven-day-old rats, caspase inhibition with therapeutic agents revealed that especially female rats were better protected in a model of unilateral focal ischemia with reperfusion [88], which was demonstrated in female mice as well [89]. These findings hold potential clinical relevance, although further mechanistic understanding is needed in order to improve female-specific clinical care standards for local recanalization and systemic thrombolysis in acute stroke settings.

## 4. Stroke Preventing or Ameliorating Mechanisms

### 4.1. Sex-Specific MicroRNAs Prevent Apoptosis of Neurons Following Cerebral Ischemia

In contrast to the aforementioned detrimental effects of X-chromosome located microRNAs, some X-linked microRNAs prevent cellular apoptosis of neurons across distinct neuronal cellular pathways, such as cellular excitation and mitochondrial respiration. Overstimulation of the glutamate receptor (glutamate excitotoxicity) is such a mechanism in neuronal cell death during stroke, because it results in abnormally high intracellular calcium concentrations. In neurons, N-methyl-D-aspartate (NMDA) receptors regulate this calcium influx, which requires membrane depolarization induced by sodium influx through 2-amino-3-hydroxy-5-methyl-4-isoxazole propionic acid (AMPAR) receptors. MiR-223 prevents NMDA-induced calcium influx, thereby protecting neurons against NMDA induced excitotoxicity and cellular apoptosis [86]. The genetic deletion of miR-223 in mice increased AMPAR subunit GluR2 and NMDAR subunit NR2B, thereby altering their composition and function [90]. In contrast, overexpression of miR-223 decreased GluR2 and NR2B expression, which inhibited neuronal NMDA-induced calcium influx and cell death following ischemia [90]. This suggests that the neuroprotective effects of miR-223 are mediated via the regulation of ion balance after ischemia.

More beneficial effects on neuronal apoptosis were seen in the MCAO model in C57/BL6 mice, which had elevated levels of the X-linked miR-424 at 1 and 4 h in the peri-infarct cortex but decreased levels after 24 h [91]. This paracrine signal reduced cellular oxidative stress, cerebral infarct size and, thereby, neuronal apoptosis. These effects were mediated with elevated antioxidant proteins because superoxide dismutase (SOD) and nuclear factor erythroid 2-related factor 2 (Nrf2) expression and activity were increased, suggesting that miR-424 protects against transient focal I/R injury by stimulating the antioxidant response [91]. Furthermore, X-linked miR-98 also has a function in reducing cellular oxidative stress. Following the MCAO model in mice, the upregulated expression of miR-98 inhibited cellular apoptosis by reducing reactive oxygen species (ROS) production and enhancing superoxide dismutase (SOD) activity in brain tissue after stroke [92]. More inhibitory effects by X-chromosome located microRNAs on the production of inflammatory cytokines and apoptosis were seen in SH-SY 5Y neuroblastoma cells (and not primary neurons although these cells contain major characteristics and function of primary neurons) following OGD in culture and in neurons upon the MCAO model in adult male C57BL/6 mice. These experimental settings led to a significant decrease in miR-18b expression whereas its gene target, annexin 3 (ANXA3, which regulates cellular inflammation and apoptosis) was increased at both mRNA and protein levels [93]. This miR decrease was also found to be neuroprotective in vitro (by promoting cell viability, decreasing cell apoptosis, reducing the production of inflammatory cytokines) and in vivo (by depressing MCAO-induced infarct size and apoptosis in mice) [93]. Interestingly, the already mentioned miR-424 also prevents this local inflammatory response by suppressing brain microglia activation leading to neuronal apoptosis. Brain microglia cells are the resident central nervous system mononuclear phagocytes [94] derived from a myeloid lineage and participate in cerebral homeostasis and the primary immune defense [95]. Microglia interact with the individual cellular components of the NVU in order to restore local homeostasis, regulate microvascular blood flow and augment cellular immunity. In vitro studies demonstrated that miR-424 overexpression inhibited microglia activity via reduced expression of microglia cycle proteins including CDC25A, cyclin D1, and CDK6 mRNA which were upregulated in brain of middle cerebral artery occlusion mice [96]. This suggests that miR-424 also regulates the cellular function of neurons indirectly via suppressing the inflammatory response of nearby microglia. More evidence for favorable outcomes with X-linked miR mediated suppression of microglia activity was found in another study into the MCAO model in mice. This study demonstrated that a higher expression of the X-linked miR-221 in neurons decreased MCAO-induced macrophage and microglia activation due to the loss of IL6, MCP-1, VCAM-1, and TNF-a mRNA and protein expression levels [97]. Whether this increased expression increases the likelihood that this regulatory control will develop differently between men and women should be studied in more detail. Compared to men, miR-221 expression is also decreased in muscle tissue in women with amyotrophic lateral sclerosis (ALS) [98], but increased in women with an isolated low HDL-C phenotype [99]. As such, altered expression of miR-221 in women could well be related to a different clinical outcome in ischemic stroke, but more studies are needed to investigate this hypothesis.

Collectively, these studies illustrate that miR-98, -223 and -424 are involved in the regulation of cellular excitation and oxidative stress in neurons following cellular ischemia. These autocrine functions may counteract the detrimental caspase-mediated cell-death pathways as described for miR-188, -223, -502 and -764 were involved. The fact that miR-223 has both roles underscores the likelihood that most miRs function pleiotropically, depending on cellular contexts and functional networks [100].

### 4.2. Sex-Specific MicroRNAs in Astrocytes Diminish Neuronal Apoptosis and Microvascular Injury

Astrocyte end feet cover cerebral capillaries in order to increase BBB integrity [101]. Moreover, bidirectional communication with neurons means that astrocyte end feet are involved in neurogenesis and synaptic function, suggesting their role in regeneration of these cells [102]. However, in OGD conditions, astrocytes start proliferating in a process called reactive astrogliosis, which is either neuroprotective (i.e., by removing excess glutamate, regulation of vascular tone and via the release of trophic factors which alters infarct volume) or a pathological cellular response [103]. Interestingly, several microRNAs transcribed from the X-chromosome participate in these biological pathways. In astrocytes from female rats but not male rats, plasma X-linked miR-363 levels were increased following MCAO at 5 days post-stroke [104]. Its expression was inversely related to infarct volume (R = −0.37, *p* < 0.05) and subsequent experiments determined that this microRNA therefore exerts neuroprotective functions, because expressed infarct volumes were higher in female rats following an antagomiR-363 injection. Interestingly, the intravenous infusion of this microRNA preserved microvascular density of female mice compared to controls and reduced the expression of caspase-3, an executioner caspase protein involved in apoptotic pathways [104]. Another study with the MCAO model in 9-month-old male mice demonstrated increased pSTAT3/STAT3 levels in ipsilateral brain tissue on days 1, 3, and 14 after cerebral I/R due to increased methylation invoked by miR-424. Mechanistic studies in cultured astrocytes demonstrated that miR-424 overexpression was shown to repress the translation of nuclear factor 1A (Nf1a, involved in astrocyte differentiation and astrogliogenesis) which induced cell cycle arrest and suppressed reactive astrocytosis which led to a preservation of neuron cellular structure in MCAO mice [105]. This suggests that miR-424 in male astrocytes and not female astrocytes prevents astrogliosis, thereby rendering those neurons more protected. Whether double dosage of this microRNA in female astrocytes similarly has neuroprotective function should be assessed in future studies. Furthermore, another neuroprotective mechanism regulated at the post-transcriptional level by microRNAs was identified after the treatment of C6 astrocytes (an astrocytoma cell line) with ischemic brain extracts (IBE) from rats which increased the expression of stromal-derived factor-1 (Sdf-1, a chemokine that regulates the injury responses in the brain which guides homing of neural progenitor cells toward damaged tissues) [106]. This elevated Sdf-1 expression enhanced the migration of neural progenitor cells and inhibited H_2_O_2_- induced cell death via the increased expression of miR-223. Mechanistically, miR-223 lowered the level of another microRNA namely miR-27b, thereby suppressing the expression of IKKα, an upstream kinase for NF-kB. Experimentally induced overexpression of miR-223 in C6 astrocytes and primary cultured astrocytes lowered miR-27b levels by suppressing the expression of IKKα, suggesting that miR-223 increased Sdf-1 expression by downregulating miR-27b [106].

In conclusion, these astrocyte-specific X-linked microRNAs exert neuroprotective functions. Given that the inhibition of miR-363 in female mice increased infarct volumes after MCAO, this suggests that sex-specific microRNA expression in astrocytes determines cerebral cellular fate in women with stroke. The fact that the coordination of caspase mediated cell death of neurons starts via X-chromosome-located miRs could increase the likelihood that this regulatory control will develop differently between men and women.

### 4.3. Sex-Specific Estrogen-Regulated MiRs in Neurovascular Units Following Cerebral Ischemia

Brain astrocytes express the estrogen receptor-α (ER-α) and -β (ER-β), thereby enabling estrogen to influence astrocyte to neuron signaling [107]. One example of this is neuronal derived estrogen that instigates astrocyte reactivity after stroke [107] and stimulates the secretion of neuroprotective, astrocyte-derived neurotrophic factors like brain-derived neurotrophic factor (BDNF) and insulin-like growth factor (IGF-1) [108]. This could mediate the many reported sex-specific astrocyte functions like the fact that female astrocytes are better protected against OGD [109] and oxidative stress-induced cellular [110] toxicity than male astrocytes.

Also, within the CNS microRNAs are influenced by estrogen levels. For instance in human studies, miR-126 correlated with both estradiol levels in serum (Spearman correlation coefficient 0.76, *p*-value 0.028) and in cerebral spinal fluid (Spearman correlation coefficient 0.65, *p*-value 0.082) [111]. However, no mechanistic experiments were performed in this study. Nonetheless, several animal studies have addressed the role of estrogen-regulated miRs following the MCAO model. In ovariectomized female mice (a surgical model of menopause), treatment with either an miR-181a antagomir or estrogen decreased MCAO infarct volume, while a miR-181-a *antagomir* alone provided a significant reduction of infarct volume, i.e., 24%, in female mice only [45]. Interestingly, this study also provided evidence for a direct target relation of ERa for miR-181a in female brain cortex and female astrocyte cultures in vitro, but not in male astrocyte cultures, suggesting a sex-specific target effect of this microRNA [45]. Next to miR-181-a, several other estrogen regulated microRNAs have been found within the peri-infarct zone of the cerebral cortex following MCAO in 12-week-old male rats. In these rats, estrogen suppletion increased the expression of miR-223 and miR-200c, (both after 6 h post-stroke) as well as miR-199 and miR-214 (both after 12 h post-stroke) [112]. Interestingly, estrogen suppletion in these male rats significantly reduced the stroke-dependent increase of miR-214 and miR-223 (with a concomitant increase of the miR-223 target genes NR2B and GRIA2, both subunits of AMPA and NMDA glutamate receptors). In contrast, miR-375 and miR-200c and its target RIPK2 were significantly increased following MCAO while estrogen increased miR-375 only [112]. Estrogen reduced the expression of the miR-200c target RIPK2 (an upstream regulator of caspase-1 and the NOD1/2 inflammatory response following ischemia), suggesting synergistic effects of estrogen and these microRNAs post-stroke with a resultant post-transcriptional regulation of pro-apoptotic and -inflammatory genes [112].

Collectively, estrogen-regulated microRNAs in astrocytes counteract sex-specific apoptotic pathways in neurons, thereby regulating infarct volume. In this way, estrogen renders women less susceptible to ischemic cell death pathways. This pinpoints the consequences of alterations in sex hormone levels which could affect stroke incidence and outcome in young women and postmenopausal women. The study by Leppert et al. [1] demonstrated a higher stroke incidence in women at a young age (24–44 years) compared to men in the same age group. This suggests either that female-specific noncardiovascular risk factors increase this incidence, as is seen with migraine, pregnancy [113], diabetes or the use of oral contraception [114], or that elevated estrogens (i.e., endogenous estrogens in combination with exogenous estrogens) may serve as a potential driver of an elevated susceptibility for ischemic stroke in women. This could alter microRNA expression as well, which was demonstrated to serve as a potential driver of an elevated susceptibility for ischemic stroke in women by affecting whole body metabolism [115]. However, more studies are needed to confirm this hypothesis.

## 5. Actual and Potential Clinical Implications

### 5.1. Biomarker Potential for Stroke Diagnosis

Clinical studies have demonstrated that sex-specific microRNAs could provide a valuable biomarker potential for the diagnosis and progression of ischemic stroke. Particularly the X-chromosome origin of plasma microRNAs may result in a sex-specific diagnostic biomarker for stroke in women. Although many studies identified the involvement of these circulating X-linked microRNAs in ischemic stroke, a stratification of circulating microRNA levels for women is oftentimes not implemented within these studies in contrast to studies into sex-specific microRNAs in female-specific cardiovascular disease pathophysiology [31]. Therefore, a meta-analysis of these data sets with stratifications of microRNA levels specifically for women and men could clarify whether measurement of these microRNAs helps to diagnose stroke in women. This could also provide additional information about whether sex-specific plasma microRNAs have a biomarker potential for silent cerebral ischemia as well, as was demonstrated previously with other microRNAs [116]. As such, these sex-specific microRNAs could potentially demonstrate a relationship between gender, silent lacunes on neuroimaging and cognitive decline [117].

One of the sex-specific circulating microRNAs involved in stroke pathophysiology is the X-linked miR-503. MiR-503 levels were positively associated with acute ischemic stroke diagnosis (OR = 11.35, 95%CI: 1.13–113.84, *p* = 0.039). Its levels were significantly increased in patients with moderate-severe stroke (*n* = 65) compared to control patients, but not in minor stroke (*n* = 67) compared to control patients [58]. Furthermore, in another study among ischemic stroke patients (*n* = 22), elevated plasma levels of the X-linked miR-505 differentiated stroke patients from controls (*n* = 22) and its levels normalized following stroke treatment [118]. Regarding stroke triggered by large or small-artery occlusion, decreased X-linked miR-221 levels were observed among patients with ischemic stroke (*n* = 46) [90]. Subsequent analysis showed that the plasma miR-221 levels were significantly decreased in patients with both large and small-artery occlusion [97]. These results were later confirmed among 20 patients with ischemic stroke (13 men and 7 women; age 35–67 years) compared to 20 healthy controls (12 men and 8 women; age 33–71 years) who presented within 72 h of the event and exhibited a NIHSS score between 4 and 15 [119]. Significantly lower miR-221 levels in ischemic stroke were further confirmed in a study among 167 subjects with ischemic stroke (*n* = 167) compared to healthy controls (*n* = 157) which demonstrated that stroke risk increased by 10-fold following the decrease of this microRNA [120].

### 5.2. Biomarker Potential for Stroke Severity and Progression

Elevated X-linked miR-223 levels were also seen in ischemic stroke patients (*n* = 79) compared to healthy controls (*n* = 75). These levels displayed a negative correlation with NIHSS scores (R = −0.531, *p* < 0.01) and infarct volume (R = −0.265, *p* = 0.039) but a positive correlation to plasma IGF-1 [121]. Because circulating miR-223 levels are significantly lower in healthy women compared to men [122] their sex-specific plasma levels of should be assessed in ischemic stroke patients as well. Furthermore, in a study among 24 ischemic stroke patients compared to healthy controls (*n* = 22), the X-linked miR-224-3p and miR-532-5p decreased following ischemic stroke [123]. Gene ontology analysis in this study revealed that particularly cellular metabolism was the top ranked biological process regulated by target genes of these decreased plasma miRs. A relation to metabolism was also identified with elevated miR-503 expression in stroke patients with diabetes compared to stroke-only patients, suggesting that both hyperglycemia and ischemia in NVUs cause overexpression of miR-503 [124]. In a similar cohort of acute stroke patients (*n* = 45), plasma levels of the X-linked miR-424 were decreased and displayed a negative correlation with inflammatory cytokines like IL-6, IL-4, and TNF-α serum levels compared to controls (*n* = 45) [125]. Similarly, when circulating inflammatory cells in plasma were isolated from acute stroke patients (*n* = 40) within 6 h of symptom onset, elevated miR-424 in lymphocytes and neutrophils associated with ischemic stroke. The neutrophil-specific expression levels in this study displayed a negative correlation with infarct volume, while lymphocyte and neutrophil miR-424 levels were both inversely correlated with plasma TNF-α, IL-10, or IGF-1 levels [126]. This suggests that these microRNAs pinpoint inflammation and metabolism as pathophysiological substrates involved in stroke progression in women. Previously, conventional stroke biomarkers have pinpointed a clear relationship between inflammation and lacunar stroke in which c-reactive protein was found to predict the risk of recurrent stroke [127]. Interestingly, this relationship between inflammation and stroke was further demonstrated in postmenopausal women [128] while a recent computational framework analysis study has emphasized an association between sex-specific microRNAs and metabolism in women [129]. This study identified sex-specific miR expression in 8 miR datasets comprising miR expression results from peripheral brain tissue, which revealed that female-specific microRNAs were involved in a higher disease spectrum width, particularly in metabolic diseases [129].

### 5.3. Biomarker Potential in Predicting the Therapeutic Response to Antiplatelet Therapy

In both the acute and chronic phase of stroke, antiplatelet therapy (aspirin and clopidogrel) is currently the first-line treatment to prevent recurrent stroke [130], albeit that the response to antiplatelet therapy differs among patients [131]. In these patients, platelet-rich plasma contains more microRNA than platelet-poor plasma and antiplatelet therapy is known to lower circulating microRNA levels [132]. This plasma microRNA response to antiplatelet therapy has been studied in further detail to assess whether microRNA levels could provide information about the response to antiplatelet therapy. This was first observed in patients with acute coronary syndrome in whom circulating miR-126 levels were found to be associated with antiplatelet therapy [133]. Interestingly, aspirin administration in patients with type 2 diabetes was found to lower circulating levels of miR-126, because it prevents the transfer of platelet-enriched microRNAs from platelets to plasma [134]. In diabetes mellitus patients with stroke, compared to diabetes mellitus only, platelets are also a major source of plasma X-linked miR-223 levels. These plasma- and platelet levels were decreased after stroke, while lower platelet levels significantly correlated with platelet reactivity and lower plasma miR-223 levels [135]. This suggests that the loss of miR-223 in platelets could serve as a risk factor for ischemic stroke in diabetes mellitus patients. Future study of this microRNA response to antiplatelet therapy will be critical to advancing our understanding of its role in predicting platelet reactivity in women.

## 6. Concluding Remarks

**Figure 2:**
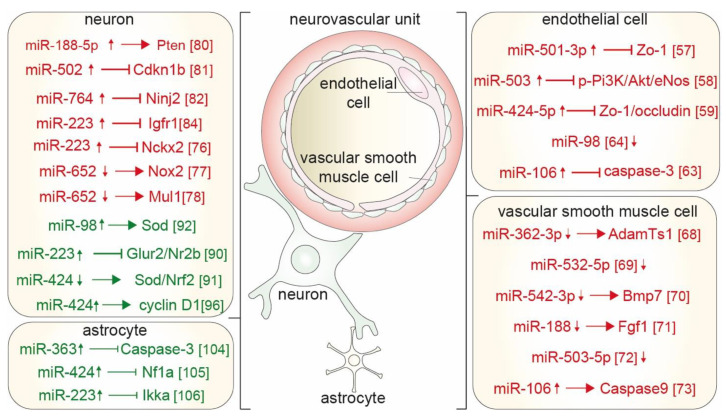
Overview of sex-specific miR expression in neurovascular units and target gene expression effect. Red font depicts stroke promoting mechanisms, while green font indicates stroke preventing mechanisms.

It has become clear that post-transcriptional networks in NVUs drive the cellular transcriptome and its response to injury [136]. As such, sex-specific noncoding RNA expression in NVUs could offer additional insights into stroke pathophysiology in women (Figure 2). Therefore, we conclude that (i) sex-specific microRNAs performed cell-type and context dependent regulation of gene expression in NVUs (i.e., elevated miR-188 in neurons promoted cellular apoptosis while decreased expression in VSMCs enhanced the proliferation of these cells); (ii) sex-specific microRNAs in vascular cells and neurons act on one another, and as such, affect the cell viability of neurons (i.e., their increased expression could promote sex-specific apoptotic pathways in neurons by promoting BBB permeability and vascular reactivity); and (iii) sex-specific microRNA expression in NVUs was often associated with the loss of NVU homeostasis, while their involvement in reactive astrogliosis served protective functions for neuronal health. Nonetheless, more human studies are needed to validate these post-transcriptional mechanistic pathways in women following stroke. This could indicate whether silencing or activation of these upstream regulators may allow for (i) a (sex-specific) biomarker that predicts the presence of silent cerebral ischemia at early stages of cerebral small vessel disease; and (ii) an effective therapeutic strategy, as was recently demonstrated following endothelium-targeted deletion of microRNA-15a/16-1 in mice [137].
